# Remarks on Wine and Alcohol

**Published:** 1871-12

**Authors:** 


*Remarks on Wine and Alcohol.* By Chas. A. Lee, M. D.

In this pamphlet the author first describes the natural order to which the vine family belongs, the process of obtaining the wine, the composition of wine, the amount of alcohol in wine, the source of the alcohol, cause of acidity of wine, the adulteration, physiological effect of wine, dietetic therapeutical use of wine. He then speaks in a similar order of alcohol. It contains very interesting and useful information and shows that the author is thoroughly familiar with the subject.

